# Comparing the effectiveness of ring and block-vaccination strategies on networks

**DOI:** 10.1371/journal.pcbi.1013274

**Published:** 2025-08-11

**Authors:** Matteo Scianna, Riccardo Gallotti, Michele Tizzoni, Oriol Artime, Lucila G. Alvarez-Zuzek

**Affiliations:** 1 Complex Human Behaviour Lab, Foundation Bruno Kessler, Trento, Italy; 2 Department of Sociology and Social Research, University of Trento, Trento, Italy; 3 Departament de Física de la Matèria Condensada, Universitat de Barcelona, Barcelona, Spain; 4 University of Barcelona Institute of Complex Systems (UBICS), Universitat de Barcelona, Barcelona, Spain; 5 Universitat de les Illes Balears, Palma, Spain; University of A Coruna: Universidade da Coruna, SPAIN

## Abstract

Vaccination is vital for preventing disease spread, as demonstrated by its role played in recent outbreaks such as measles, COVID-19, and the 2014 West Africa Ebola crisis. Classical ring vaccination–targeting individuals near infected cases to form protective clusters–has become of interest due to its effectiveness, yet it is strongly influenced by the quality of contact tracing and availability of medical resources. Here, we model the efficiency of a family of ring vaccination-inspired strategies that address these limiting factors and disentangle them from the structure of the contact patterns. In particular, we evaluate scenarios that consider a vaccination radius *r*, used to vaccinate nodes in the network up to *r* contacts away (*block vaccination*) or exactly *r* contacts away (*ring vaccination*) from nodes of interest. Each one of these is tested under two further scenarios: the *preventive* one, where individuals are vaccinated before the epidemic takes place, and the *containment* one, where vaccination occurs during an outbreak to limit disease spread. They are tested in synthetic networks, where we find that in the preventive scenario, ring outperforms block vaccination, reducing the size of the epidemic and, in some cases, even preventing it from happening. On the other hand, in the containment scenario, we find that both strategies perform slightly similarly in reducing the impact of the diseases but block vaccination using fewer resources. As a case study, the proposed strategies are used to create epidemiological risk maps by employing the spatial position of olive trees in the Salento region in Italy, which recently suffered the impact of the bacterium *Xylella fastidiosa*.

## Author summary

Infectious diseases such as measles and Ebola have shown how quickly outbreaks can spread and how essential vaccination is to protect individuals. In our work, we investigate a spectrum of vaccination strategies, inspired by ring vaccination, that can help to contain the spread of epidemics by targeting individuals in close contact with confirmed cases. We explore two scenarios: a *preventive scenario*, where vaccines are administered before an outbreak begins, and a *containment scenario*, where vaccines are used to mitigate the spread of a disease that is already underway. We develop mathematical frameworks to describe the proposed models, utilizing synthetic complex networks as a proxy to mimic social contact patterns. We found that, depending on timing and strategy, these targeted approaches can be very effective, even stopping outbreaks entirely or reducing their impact while using fewer vaccine resources. To illustrate the performance and impact in a real-world setting, we framed our models within the context of olive trees in southern Italy, where a harmful bacterium has been spreading over the last decade. Our results suggest that such vaccination models could provide tools to prevent and face epidemic diffusion in a wide range of scenarios, from public health to agricultural decisions.

## Introduction

Epidemic spreading is at the core of many complex phenomena [1] spanning a wide range of spatial and temporal scales [2,3], from a microscopic level with virus dynamics infecting cells [4] up to a macroscopic level, with behavioral societal effects such as infodemics [5–7] and vaccination hesitancy that generates spatial heterogeneity of documented undervaccination [8–12]. Controlled experiments in epidemiological contexts are infeasible; therefore, approaches based on mathematical modeling have become very valuable to the scientific community in helping address public health issues. In particular, specific models can be used to evaluate epidemic scenarios under different intervention strategies. In addition, human interactions can change the future course of an epidemic outbreak. The structure of social patterns can be represented by complex networks [13], typically characterized by highly heterogeneous and non-trivial connectivity relations, such as modular and hierarchical structure [14], and multilayer [15] and higher-order [16,17] interactions.

On these bases, great attention has been devoted to unraveling the role of the network structure in the spread of an epidemic [18]. Mathematical frameworks, such as percolation theory and message passing, have become commonplace in this field [19–22,56], allowing researchers to explore the impact of vaccination strategies as a function of the underlying network topology [23]. For instance, it is well known that, for heterogeneous connectivity patterns, the epidemic threshold can become arbitrarily small and even vanish if individuals with a large number of connections (hubs) are present [24]. However, one can still take advantage of heterogeneous connectivity patterns to deploy vaccination strategies that are more effective than random mass vaccination, which turns out to be especially relevant in scenarios with limited resources. This includes exploiting information related to the centrality of individuals in the social network, along with their age group, socioeconomic status, level of education, and medical records [25–27].

Vaccination is the most effective intervention to mitigate the burden of infectious diseases. Achieving optimal levels of vaccination close to herd immunity results in sharp decreases in morbidity and mortality and, in some cases, allows for the eradication of infectious diseases [28]. An efficient vaccination strategy that relies heavily on the connectivity patterns of infected individuals is the so-called ring vaccination [29,30]. Since the contacts of an infected individual are at high risk of transmission, the strategy focuses on vaccinating first contacts and, in some cases, individuals in close contact with those first contacts. The aim is to create immunity buffers around new cases, a bubble of immunity around infected individuals that impedes further diffusion beyond local infection. Ring vaccination has proven to be highly effective when traditional approaches are not feasible and in limited resource settings [30]. For example, during the 2014−−2016 West African Ebola epidemic, the *Ebola ça suffit* ring vaccination trial was developed and applied to evaluate the rVSV-ZEBOV Ebola vaccine, successfully mitigating the outbreak [31]. From a theoretical perspective, researchers have developed mathematical frameworks to understand and analyze ring vaccination on synthetic network structures—single and multilayer—and how limited resources impact the dynamic [32–34]. From a computational point of view, some large agent-based, data-driven models have been proposed [27,35]. Although the use of contact rings can be a highly effective strategy, understanding its effectiveness is not trivial, mainly due to the difficulties associated with real cohorts and their contact patterns [36]. Contact tracing can be determinant in controlling and eliminating a disease [37,38], yet it may be complex to carry out. This complexity increases further if implemented during an ongoing pandemic, as was the case with COVID-19, where several factors may reduce its effectiveness, including delays in processing test results, inadequate staffing, and challenges in locating contacts [39]. Therefore, despite the successful applications of ring vaccination, several important aspects remain unexplored, resulting in an incomplete understanding of this vaccination strategy and its potential for enhancing performance. In real cohorts, unreported contacts may facilitate uncontrolled pathways of infection. Does considering vaccination contacts that are more than one hop away from the index case make the strategy more efficient? What if only a fraction of the ring is vaccinated? Is there a threshold for the trade-off between performance and resources? To deepen our understanding of ring vaccination strategies, it is crucial to have a bare-bones mathematical model that can analytically unravel the qualitative relationship between the network properties and key epidemiological quantities.

**Fig 1:**
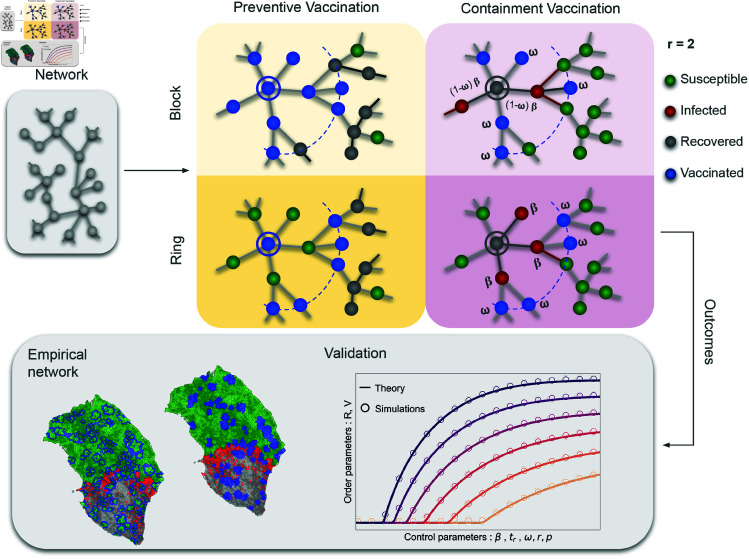
Description of the vaccination dynamics. We apply the strategy to the same original contact pattern network to study the different vaccination dynamics. As a first differentiation, we consider i) *Preventive scenario*: in which the vaccination dynamic occurs before the appearance of the zero patient, leading the disease to propagate in a diluted original network; ii) *Containment scenario*: where the vaccination dynamic occurs simultaneously with the spreading process, every time new infected individuals appear a set of their neighbors are chosen to be vaccinated. Besides, we explore the cases of (un)limited resources: a) *Block vaccination*: in which all the individuals within the ring are vaccinated; and b) *ring vaccination*: where resources are limited hence, only individuals in the largest radio (border of the ring) are vaccinated. In the colored panels, we display a one-time step of the different vaccination dynamics considering a radio of vaccination of second neighbors (*r* = 2). For the propagation of the disease, we consider a modified version of the *SIR* model where individuals can be in one of four different states: Susceptible (green), Infected (red), Recovered (gray), or Vaccinated (Blue). Yellow panels correspond to the asymptotic state of the preventive scenario (after the disease propagates, without infected individuals). Islands of susceptible (ring) or vaccinated (block) individuals appear depending on the strategy, shaping the reach of the epidemic. Pink panels correspond to an intermediate step of the disease propagation. Conversely, in this case, for every newly infected individual, with a certain probability, a portion of their neighbors will get vaccinated with the intent of breaking the chain of infection. We develop a mathematical framework for this family of vaccination strategies and validate our results with stochastic simulations. Through a case study, we show how to build maps of epidemic risk in spatially embedded networks. Data for the creation of the maps taken from: https://www.istat.it/notizia/confini-delle-unita-amministrative-a-fini-statistici-al-1-gennaio-2018-2/.

The remaining of the article is organized as follows. In the results section, the theoretical approaches behind the proposed vaccination strategies are presented, see Fig 1. We introduce the main quantities of interest, show how to compute them, and verify the validity of the theory. Secondly, we offer epidemiological insights learned from both our mathematical treatment and computer simulations. Finally, we investigate the application of the proposed vaccination strategies in an empirical scenario of olive trees located in southern Italy, which recently suffered the impact of the bacteria *Xylella fastidiosa*. In the Discussion section, the main results are analyzed and contextualized. Structural differences between the proposed models are also highlighted and discussed in terms of their performance and effectiveness in preventing epidemic diffusion. Then, the Methods section contains a detailed description of the theoretical equations of the dynamics. We close the article by drawing conclusions.

## Results

### Theoretical approaches toward different ring vaccination strategies

In this section, we present the main quantities used to characterize each vaccination strategy and validate them in representative ensembles of network topologies. The details of the derivations are left to the Methods section. Subsequent sections exploit these results to discuss epidemiological insights regarding the efficiency of each strategy. For the evaluation of efficacy, we focus on two main quantities: in the preventive scenario, equations allow us to study the capability of the vaccination model to dismantle the network, consequently avoiding long chains of infections; in both the preventive and containment scenarios, we further focus on the final size of the epidemic as the key quantity to be minimized, depending on both the probability of infection and the probability of vaccination.

#### Preventive scenario: Static vaccination.

In this preventive vaccination scheme, we decouple the vaccination and spreading processes. We first vaccinate the nodes according to the block or ring rules we have outlined above (see also Fig 1), to afterward evaluate the effectiveness of the vaccination strategy by running the epidemic model on top of the unvaccinated population. We have some freedom in choosing the epidemic model for which we are going to test the strategy effectiveness, so, without loss of generality and for illustrative purposes, we take here the classical Susceptible-Infected-Recovered (*SIR*) model [40]. In Table 1 there is a summary of the parameters used for this dynamic.

**Table 1:** Summary of parameters occurring in the proposed equations - Preventive scenario.

Symbol	Meaning
*ϕ*	fraction of vaccinated nodes
*N*_*t*_	Size of the network at time *t*
*p*_*t*_(*k*)	time-dependent degree distribution
*q*_*t*_(*k*)	time-dependent excess degree distribution
$\langle k\rangle_{t}$	Expected value of *p*_*t*_(*k*)
$G_{0}^{t}(x)$	Generating function of the degree distribution at time *t*
$\Omega_{t}^{r}$	Expected number of nodes vaccinated for radius *r* at time *t*
*s*_*t*_	Size of the Giant component at time *t*

Initially, we assume individuals to be in either of two states: susceptible *S* or vaccinated *V*. As is usual in percolation approaches applied to epidemiological contexts, susceptible individuals *S* are interpreted to be present in the network and potentially participate in the epidemic dynamics, whereas *V*-nodes are considered removed and do not play an active role in spreading the disease. Our goal in this part of the research is to compute the resulting topology formed by *S*-nodes after the vaccination, which results in the contact structure on top of which the disease can spread. In particular, we focus on the time-dependent giant component *s*_*t*_ of susceptible nodes. Intuitively, the giant component of a network is the largest connected cluster (component) that contains a non-negligible proportion of the total number of nodes. We study how the proposed vaccination strategies shape the evolution of such a quantity. This is chosen as a proxy to understand the efficiency of the vaccine: the lower *s*_*t*_ after vaccination, the less damage a disease can induce. Therefore, efficient vaccination strategies are those that lead to small sizes of the giant component of *S*-individuals, breaking the potential long chains of infection and curbing the reach of the diseases.

A static vaccination step of radius *r* is divided into two parts, which we mathematically describe in separate ways. First, a node *i* is randomly selected among those susceptible, i.e. those not vaccinated in previous steps. Then, we select the sample of the nodes to vaccinate according to the block or the ring cases. In the former, all the nodes that are up to a distance *r* away from our central node *i* are chosen, while in the latter, only the corona composed of nodes exactly *r* hops away is selected. These two steps (random selection of susceptible node and vaccination of the corresponding neighbors) are repeated until a fraction *ϕ* of vaccinated nodes is reached. The parameter *ϕ* can be interpreted as the accessibility of resources, *e.g.*, the number of vaccines available to immunize the population. In classical percolation, the parameter *ϕ* is the occupation probability, and it is the control parameter of the percolation phase transition. Here, in an epidemiological context, we expect as well a phase transition from an epidemic to a non-epidemic phase mediated by *ϕ*.

We write *p*_*t*_(*k*) as the time-dependent probability degree distribution of the network, namely the probability that an unvaccinated node has *k* connections at time *t*. The goal next is to obtain $p_{\phi }(k)$, the degree distribution of the resulting network of non-vaccinated *S*-nodes when a fraction *ϕ* of nodes has been vaccinated. This is to later exploit the well-established mapping between the *SIR* model and bond percolation [19,34]. By employing generating function techniques, which depend on the degree distribution, we will evaluate the effectiveness of vaccination strategies and the impact of the epidemics. Let us define, for convenience, a discrete “time” variable $t\equiv N_{0}\;\phi$, where *N*_0_ is the initial size of the network. Notice that the first part of the vaccination step –random selection of the central susceptible node and its vaccination– is the same for the block and the ring strategies. We show in the Methods section that in this first part, the time-dependent degree distribution of the network varies according to

$$p_{t}(k)=\frac{1}{N_{t}}\left[p_{t-1}(k)N_{t-1}-p_{t-1}(k)+\langle k\rangle_{t-1}\left[q_{t-1}(k)-q_{t-1}(k-1)\right]\right],$$ (1)

where *t*–1 and *t* refer to the arbitrary times before and after vaccinating the central node, respectively. *N*_*t*_ is the number of unvaccinated nodes in the network when a fraction of *ϕ* nodes has been vaccinated, i.e., $N_{t}=N_{0}\;-\;t$, *q*_*t*_(*k*) is the time-dependent excess degree distribution and $\langle k\rangle_{t-1}$ is the expected value of *p*_*t*−1_(*k*). After the vaccination of the central node, the evolution of the degree distribution depends on the type of strategy implemented. We particularize each case in the following.

For static block vaccination, the generating function of the degree distribution of the network after the vaccination process takes place reads

$$G_{0}^{t+\Omega_{t}^{r}}(x)=\frac{1}{G_{0}^{t}(f)}G_{0}^{t}\left[f+\frac{\left(G_{0}^{t}{)}^{\prime }(f\right)}{\left(G_{0}^{t}{)}^{\prime }(1\right)}(x-1)\right],$$ (2)

where $f\equiv {\left(G^{t}\right)}_{0}^{-1}(\phi )$ and $\Omega_{t}^{r}$ is the number of nodes that will be radially vaccinated after the root node is chosen at time *t*. See the Methods section for details. This quantity depends non-linearly on the first and second moment of the time-dependent degree distribution (1). Yet, using that $G_{0}^{t}(x)=\sum_{k}p_{t}(k)x^{k}$, Eq (2) can be inverted numerically, hence the degree distribution after one block vaccination step is available. We can iterate the procedure encoded in Eqs (1)–(2) and we readily obtain the degree distribution after an arbitrary number of nodes has been vaccinated.

Regarding the case of static ring vaccination, the derivation is simpler, yet at the expense of more drastic assumptions. In the Methods section, we show that the degree distribution can be approximated by

$$p_{t+\Delta \Omega_{t}}(k)=\frac{n_{t+\Delta \Omega_{t}}(k)}{\sum_{i=1}^{k_{\text{max}}}n_{t+\Delta \Omega_{t}}(i)},$$ (3)

where $\Delta \Omega_{t}\equiv \Omega_{t}^{r}-\Omega_{t}^{r-1}$, $k_{\text{max}}$ is the maximum degree of the network at time *t* and $n_{t+\Delta \Omega_{t}}(k)$ is the number of nodes with degree *k* at time $t^{\prime }=t+\Delta \Omega_{t}$,


$$n_{t+\Delta \Omega_{t}}(k)=\left\{ \begin{array}{cc} p_{t^{\prime }}(k)\cdot N_{t^{\prime }}, & k>0\\ p_{t^{\prime }}(k)\cdot N_{t^{\prime }}+\Omega_{t}^{r-1}, & k=0. \end{array}\right.$$

Using this time-dependent degree distribution, we readily have access to the time-dependent generating functions of the static ring vaccination process.

Combining the pieces, the relative size of the giant component of unvaccinated nodes at time *t*, *s*_*t*_, is given by the coupled system of equations

$$\left\{ \begin{array}{c} u_{t}=G_{1}^{t}(u_{t})\\ s_{t}=1-G_{0}^{t}(u_{t}), \end{array}\right.$$ (4)

where $G_{1}^{t}(x)=\partial_{x}G_{0}^{t}(x)/\langle k\rangle_{t}$ is the time-dependent probability generating function of the excess degree distribution. We need to insert in Eqs (4) the generating function (2) when dealing with block vaccination and the ordinary generating function $G_{0}^{t}(x)\equiv \sum_{k}p_{t}(k)x^{k}$, with *p*_*t*_(*k*) given by Eq (3), for the ring vaccination case. It is difficult to have analytical insights directly from Eqs (4), but they can be solved numerically by first finding the values *u*_*t*_ and then plugging them into $G_{0}^{t}(u_{t})$ to find the relative size of the giant component. For the relevant cases where the initial network is connected, *u*_*t*_<1 holds and *s*_*t*_>0. As the vaccination processes take place, there will be a critical time *t*_*c*_ where $u_{t_{c}}=1$ will be the only solution to the first equation in (4), and $s_{t\geq t_{c}}=0$. This means that *t*_*c*_ represents an upper bound for the minimum number of nodes that need to be vaccinated for a disease outbreak not to become an epidemic. Notice that we are under the assumption of a vaccine efficacy of 100%.

**Fig 2:**
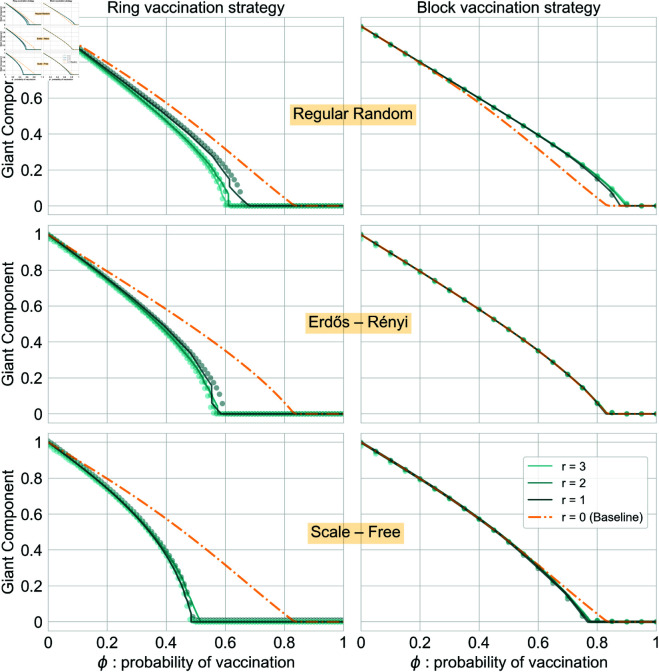
Validation of the theoretical framework for the case of Preventive—static—scenarios. For the cases of ring (left panel) and block (right panel) vaccinations. The radius of vaccination spans from 0 (baseline that corresponds to random vaccination) to 3. As the vaccination strategy occurs before the propagation of the disease, we validate our results by analyzing the evolution of the giant component as a function of the percentage of vaccinated nodes *ϕ*, *i.e.,* the resulting diluted network after the percolation process. Each horizontal panel corresponds to the same network structure with *N* = 10^4^ nodes, ranging from more homogeneous on the top, a random regular graph with ⟨k⟩=6, to more heterogeneous on the bottom, a scale-free network with degree exponent γ=2.5 and $k_{\text{min}}=3$ and $k_{\text{max}}=20$. In the middle, results are shown for Erdős-Rényi nets with ⟨k⟩=6. See the Methods Section for details further details on these network ensembles. Experimental results are averaged over 10^3^ independent stochastic simulations.

We validate the accuracy of our mathematical frameworks for the preventive vaccination scenario strategies in Fig 2, finding an excellent agreement between theoretical and simulated results. The general trend is that ring outperforms block vaccination, and this is robust for all the parameters considered. For the same amount of vaccine supplies used, ring vaccination is more efficient, resulting in lower sizes of the giant component of *S*-nodes, *i.e.*, smaller pools of susceptible individuals that can be vulnerable to eventual epidemic outbreaks. Besides, in this scenario, the critical fraction of vaccinated individuals $\phi_{c}$ is lower, meaning that fewer vaccine resources are needed to fragment the network to the point that an epidemic cannot develop. We can also see from Fig 2 that ring vaccination is always a better strategy than vaccinating randomly (baseline). However, this is not the case for block vaccination, where, for more homogeneous topologies, it is better to vaccinate randomly, while for heterogeneous topologies, the performance is reversed. Note that this result is consistent with the results presented in [41]. Furthermore, by comparing the threshold when vaccination is applied with the baseline model, we can see the important role degree heterogeneity plays in the process. In both cases, moving from more homogeneous to more heterogeneous topologies (from top to bottom panels), we observe a decrease in $\phi_{c}$. This trend is stressed in the block strategy: while, as already stressed in [41], for very homogeneous networks such as RR, the block vaccination strategy performs worse than a random approach. We see a progressive outperformance concerning the baseline curve. Finally, we can see that vaccination radius *r* plays a less important role in the block vaccination case, as the giant component *s*_*t*_ nearly collapses for the explored radii r≠0. In the case of ring vaccination, the effect of varying radii is still subtle but more appreciable. Notice that, as already stated, when *r* becomes larger than 3 the coverage of vaccinated individual is so extensive that its overall impact stabilizes. Specifically, given the structure of the most contact patterns, where the small-world effect predominates and the number of connections is distributed as a power-law, choosing larger values of *r* is neither convenient nor empirically relevant. One reason is that greater values of radius would lead to select too many nodes at once, making the vaccination process unrealistic. (The number of nodes in each layer grows exponentially). Similarly, the average distance between any pair of nodes, the so-called average path length, increases logarithmically with the number of nodes in the network. Hence, vaccination radii cannot be very larger since they would cover the entire network in one single vaccination event.

#### Containment scenario: Dynamic vaccinations.

We move now to the dynamic case scenario, where the vaccination process takes place simultaneously with the spreading of the epidemic. As for the previous section, all quantities are summarized in Table 2. At each step of the process, a node can be in any of 4 states, namely, susceptible (*S*), infected (*I*), recovered (*R*), and vaccinated (*V*). $q_{i}^{X}(t)$, with X∈{S,I,R,V}, denotes the probability that node *i* is in state *X* at time *t*. The process can be seen as an *SIR*-*V* model, for which knowing the value of $q_{i}^{X}(t)$ for all the nodes of the network at all times allows us to obtain a complete view of the evolution of the epidemics and the vaccination process. Note that, at odds with the preventive scenario, in dynamic vaccination, we cannot directly control the number of vaccinated nodes, and the time *t* will have a completely different physical meaning. Let us call an *r*-neighbor a node in the *r*-th layer and $\partial^{r}$ the set of the *r*-neighbors of a certain node. At each time step of the dynamics, a susceptible node becomes infected by an infected 1-neighbor with probability *β*, while infected nodes recover at each discrete time step with probability *γ*. These two transitions S→I and I→R are the same for the block and ring cases; the difference lies in how the vaccination transition S→V is implemented. Similar to the static scenario, we investigate each case separately.

**Table 2:** Summary of parameters occurring in the proposed equations — Preventive scenario.

Symbol	Meaning
$q_{i}^{I}(t)$	Probability that node *i* is in state *I* at time *t*
$q_{k\to i}^{I}(t)$	Probability that node *k* is in state *I* at time *t* when node *i* is absent from the network
$\theta_{k\to i}(t)$	Probability that the infection has not been passed from *k* to *i* up to time *t*
$\delta_{k\to i}(t)$	Probability that the vaccination has not been passed from *k* to *i* up to time *t*
*β*	Infection probability
*γ*	Recovery probability
*ω*	Vaccination probability

We start with the dynamic ring vaccination scenario. Here, susceptible nodes become vaccinated with probability *ω* if they have an infected *r*-neighbor. This can be interpreted, for instance, as the action of a public health administration that performed contract tracing and proposes the vaccination of indirect contacts. Thus, the probability *ω* can be related to the efficiency of the contract tracing or the vaccination hesitancy of the *r*-neighbor. We employ a dynamic message-passing approach to model the state transitions. This technique lends its name to the interpretation of seeing the infection and the vaccination processes as messages propagating from infected to susceptible nodes. We show in the Methods section. that the time evolution of the probabilities of the nodes belonging to each compartment follows, for the ring vaccination case,

$$q_{i}^{S}(t)=q_{i}^{S}(0)\cdot \prod_{k\in \partial i}\theta_{k\to i}(t)\cdot \prod_{k\in \partial^{r}i}\delta_{k\to i}(t)$$ (5)

$$q_{i}^{I}(t)=q_{i}^{I}(t-1)\cdot (1-\gamma )+q_{i}^{S}(0)\cdot \Delta \theta_{k\to i}(t)\cdot \prod_{k\in \partial^{r}i}\delta_{k\to i}(t)$$ (6)

$$q_{i}^{R}(t)=q_{i}^{R}(t-1)+q_{i}^{I}(t-1)\cdot \gamma$$ (7)

$$q_{i}^{V}(t)=1-q_{i}^{S}(t)-q_{i}^{I}(t)-q_{i}^{R}(t),$$ (8)

where

$$\theta_{k\to i}(t+1)=\theta_{k\to i}(t)-\beta \cdot \phi_{k\to i}(t)\;\delta_{k\to i}(t+1)=\delta_{k\to i}(t)-\omega \cdot \phi_{k\to i}(t)$$ (9)

respectively refer to the probabilities that the infection and the vaccination messages have not been passed from node *k* to node *i* up to time *t*. $\Delta \theta_{j\to k}(t\;+\;1)\equiv \prod_{j\in \partial k⧵i}\theta_{j\to k}(t)\;-\prod_{j\in \partial k⧵i}\theta_{j\to k}(t\;+\;1)$ is the probability that the infection message actually passes between *t* and t+1 from *k* to any of the *r*-neighbors of *k*. $\phi_{k\to i}(t)$ is the probability that no message passed from node *k* to node *i* up to time *t* and node *k* is in the state in which it can pass the message to node *i* (in our case, *k* being infected). The latter can be computed recursively as,

$$\phi_{k\to i}(t+1)=\phi_{k\to i}(t)\cdot (1-\beta )\cdot (1-\gamma )\cdot (1-\omega )\;+q_{k\to i}^{S}(0)\cdot \Delta \theta_{j\to k}(t+1)\cdot \prod_{j\in \partial^{r}k⧵i}\delta_{j\to k}(t+1).$$ (10)

See the Methods section for a detailed derivation and interpretation of these quantities. We can easily re-adapt the message-passing equations of the ring case in order to obtain those of the block case. Instead of solely multiplying over the contribution of the *r*-neighbors, we also need to take into account all nodes of the intermediate shells. Thus, we find

$$\phi_{k\to i}(t+1)=\phi_{k\to i}(t)\cdot (1-\beta )\cdot (1-\gamma )\cdot (1-\omega )\;+q_{k\to i}^{S}(0)\cdot \Delta \theta_{j\to k}(t+1)\cdot \prod_{\rho =1}^{r}\left(\prod_{j\in \partial^{\rho }k⧵i}\delta_{j\to k}(t+1)\right).$$ (11)

The time-dependent state probabilities for the dynamic block vaccination scenario follow,

$$q_{i}^{S}(t)=q_{i}^{S}(0)\cdot \prod_{k\in \partial i}\theta_{k\to i}(t)\cdot \prod_{\rho =1}^{r}\left(\prod_{k\in \partial^{\rho }i}\delta_{k\to i}(t)\right)$$ (12)

$$q_{i}^{I}(t)=q_{i}^{I}(t-1)\cdot (1-\gamma )+q_{i}^{S}(0)\cdot \Delta \theta_{k\to i}(t)\cdot \prod_{\rho =1}^{r}\left(\prod_{k\in \partial^{\rho }i}\delta_{k\to i}(t)\right).$$ (13)

The equations for $q_{i}^{R}(t)$ and $q_{i}^{V}(t)$ remain unchanged.

**Fig 3:**
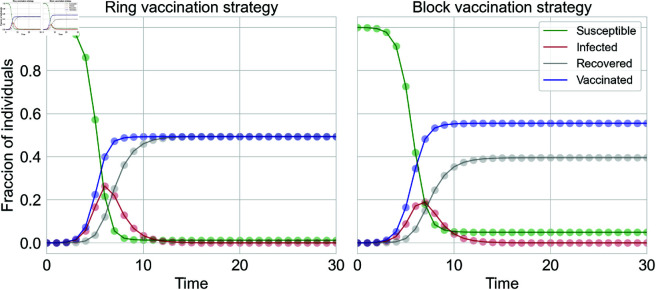
Validation of the theoretical framework for the case of Containment—dynamic—scenario. For the cases of ring (left panel) and block (right panel) vaccination. The radius of vaccination considered is *r* = 2. As the vaccination occurs along with the propagation of the disease, we validate our results by analyzing the evolution over time of the individuals in each compartment of the *SIR-V* model. The epidemiological parameters values are β=1, γ=1 and ω=0.15, and the process occurs on top of a Scale-Free network with λ=2.5, $k_{\text{min}}=3$ and $k_{\text{max}}=20$, and size *N* = 10^4^. Experimental results are averaged over 10^3^ independent stochastic simulations.

The validity of the equations is verified in Fig 3. We report an excellent agreement between theoretical and simulated results. The message-passing techniques for the dynamic employed here give the probabilities at a node level. To obtain aggregated, network-wide macroscopic information, we average, over all nodes, the probability of being in each state. In this containment vaccination campaign scenario, the qualitative results obtained for static prevention are slightly different; specifically, block vaccination provides an advantage in reducing the impact of the disease in the population compared to the ring vaccination strategy. This can be observed by examining either the asymptotic value of recovered individuals or the height of the peak infected population. Both metrics are approximately 20% smaller in the block vaccination case than in the ring case.

### Impact of the vaccination campaigns on the epidemic reach

In this section, we investigate in detail the impact of the four proposed vaccination campaigns on the population as a function of the corresponding epidemiological parameters and the vaccination radius. We consider the asymptotic percentage of recovered individuals (*R*) as an indicator of the model’s ability to effectively reduce the impact of the disease.

**Fig 4:**
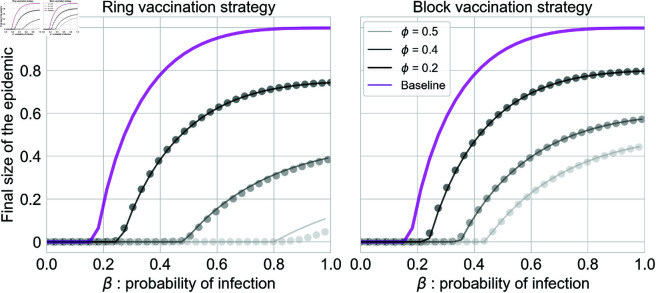
Asymptotic size of the epidemic (total fraction of recovered individuals R) as a function of the virulence of the diseases *β* for the preventive scenario. Solid lines are theoretical predictions, and symbols correspond to stochastic simulations. Grey lines correspond to the case with vaccination strategies, while violet lines correspond to the standard *SIR*. The initial size is *N* = 10^4^, and the results are the average computed over 10^3^ realizations. We consider scale-free networks with exponent γ=2.5, $k_{\text{min}}=3$ and $k_{\text{max}}=20$. The recovery probability is set in γ=1, and the vaccination radius is equal to *r* = 2. Different lines refer to different initial percentages of vaccinated nodes *ϕ*.

In Fig 4, we display the results for the preventive scenario. As a general trend, we confirm what it has been reported in Fig 2, namely, that ring vaccination outperforms the block strategy. On the one hand, in the ring case, the epidemic threshold $\beta_{c}$ (value above which the disease impacts a macroscopic fraction of the population) is always higher than block vaccination’s $\beta_{c}$. This means that more virulent diseases are necessary to become an epidemic, and this is consistent for all values of *ϕ*. For instance, by focusing on a fraction of vaccinated individuals ϕ=0.4, $\beta_{c}\approx 0.5$ for block vaccination, while for ring vaccination ≈0.35. On the other hand, for the same set of (*β*, *ω*) parameters, the asymptotic epidemic size is always lower in the case of ring vaccination. For instance, in the case β=1, which is the worst possible epidemic scenario where everyone in contact with the disease gets infected, we can observe a 10% difference in the epidemic size.

**Fig 5:**
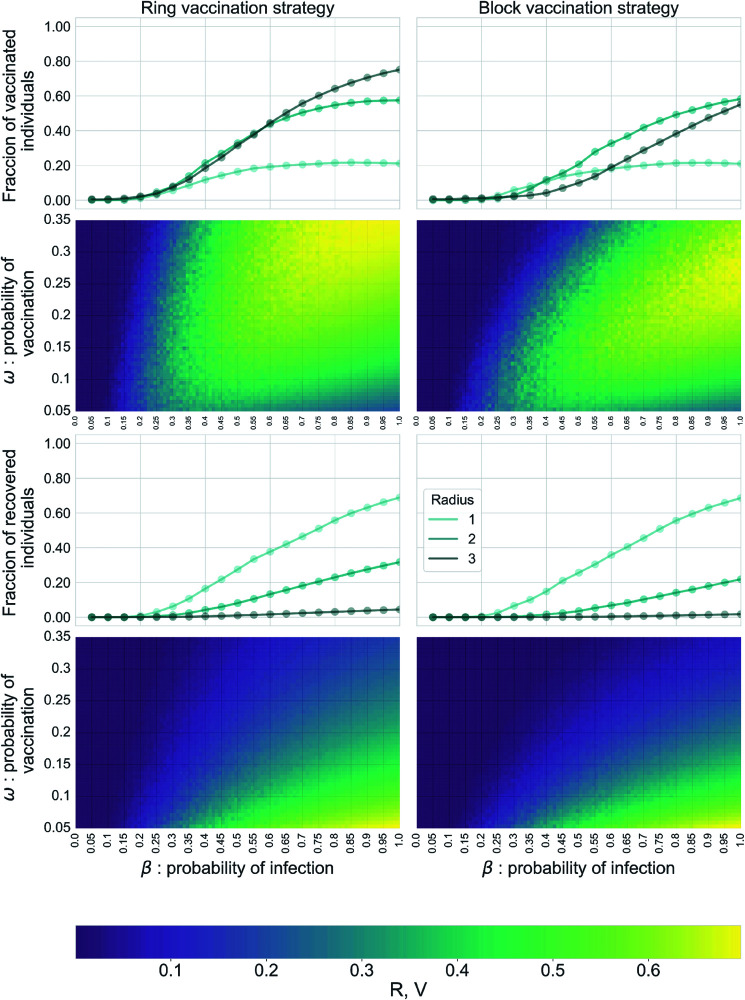
Epidemic impact in the containment scenario. We consider scale-free networks with an exponent γ=2.5, $k_{\text{min}}=3$ and $k_{\text{max}}=20$, and size *N* = 10^4^. The recovery probability is set in γ=1. **1st and 3rd rows** display the fraction of vaccinated (top) and recovered (bottom) individuals at the asymptotic state of the epidemic. Each line corresponds to different values of vaccination radius, r=1,2,3, with a probability of vaccination ω=0.25. Panel on top. Dotted lines are theoretical predictions, and symbols correspond to stochastic simulations. **2nd and 4th rows**: Heatmaps show the dependence of the vaccinated (top) and recovered (bottom) individuals on both the probability of vaccination *ω* (*y*-axis) and the virulence of the disease *β* (*x*-axis) for the specific case of *r* = 2.

We now move to the containment scenario case, i.e., vaccination co-evolves with the diseases. Our control parameters here are the probability *ω* of vaccinating a neighbor of an infected node, and the virulence of the disease *β*. We present the results in Fig 5. On the one hand, we show the asymptotic value of the fraction of vaccinated (1st row) and recovered (3rd row) individuals as a function of *β* and fixed *ω*, for different radii of vaccination. On the other hand, the dependence of these asymptotic values on *β* and *ω* for a fixed radius of vaccination is displayed as heatmaps (2nd and 4th rows, respectively).

By focusing only on the evolution of recovered individuals (3rd row), we do not notice a significant difference between the two vaccination protocols: only for the case of *r* = 2 there is a slightly lower percentage of recovered individuals for block vaccination, while for the other two values of *r*, the curves are almost identical. Nonetheless, when referring to the percentage of vaccinated (1st row), we highlight how the same amount of recovered nodes demands fewer resources, especially for higher values of *r*: for *r* = 2, we can see that the curves of vaccinated individuals are almost overlapping, but the correspondent value of recovered ones is lower of a 0.1 factor in the block strategy. This behavior is also confirmed by the other values of vaccination probability *ω*: by looking at the heatmaps, we can observe how the values of *R* (heatmap at the bottom) do not differ between the two techniques, but fewer vaccines are needed on the right column (heatmap in the middle), *i*.*e*. the heatmap for block vaccination has a greater darker area. All in all, by analyzing Fig 5, we conclude that even though both strategies lead to similar epidemic outcomes, block outperforms ring vaccination in terms of the use of resources.

### The spread of *Xylella fastidiosa* on a spatial network of olive trees as a case of study

So far, we have analyzed the case of an epidemic spreading on synthetic network ensembles characterized by the lack of correlations. Hence, a natural step forward is to explore the impact of the vaccination strategies on real network topologies. To this aim, we leverage a geotagged dataset [42] that contains the position of more than 60000 olive trees in the Salento sub-region of Apulia, Italy (see the Methods section for more details). Since 2008, an ongoing outbreak of a wasting plant disease called *Olive quick decline syndrome* has infected more than 21 million olive trees in the region, leading to widespread agricultural damage. The main cause of the disease is a strain of the bacterium *Xylella fastidiosa*, which is spread by plant-sucking insects. Already analyzed and studied in similar epidemiological contexts [43], such an epidemic represented, and still does, a serious threat to the local economy and the biodiversity of this region [44].

**Fig 6:**
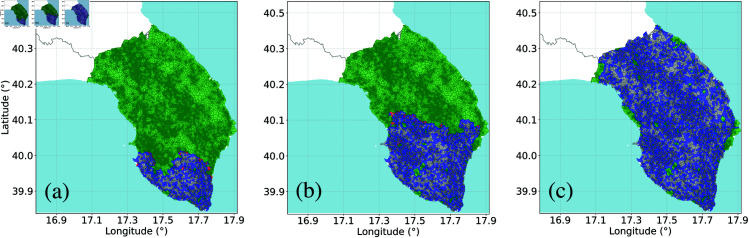
Impact of block vaccination strategy for a containment scenario on the olive tree empirical network: evolution of the diseases. Snapshots of the advance of the disease at different times, spanning from the early stages of the propagation (A) to the asymptotic state when there is no more presence of infected trees (C). Single nodes are colored according to their state during the process: Susceptible (green), Infected (red), Recovered (gray), and Vaccinated (blue). The epidemiological parameter values are: probability of infection β=1, probability of recovery γ=1, and probability of vaccination ω=0.2. Data for the creation of the maps taken from: https://www.istat.it/notizia/confini-delle-unita-amministrative-a-fini-statistici-al-1-gennaio-2018-2/.

An interesting characteristic of spatial networks is that they allow for a direct visual representation of disease propagation, vaccination dynamics, and their interaction. Fig 6 shows three different steps of the containment block vaccination process, while we refer to Fig B, Fig C, and Fig D in S1 File for the figures describing other vaccination scenarios. In the particular context of the *Xylella fastidiosa*, both *R*-trees and *V*-trees are considered dead, but for different reasons: in the former, the tree dies due to the infection caused by the bacterium, while in the latter, it dies as a result of human interventions. Indeed, since no cure or vaccine is currently known, uprooting the trees is the main practice to hinder the spread of the disease.

**Fig 7:**
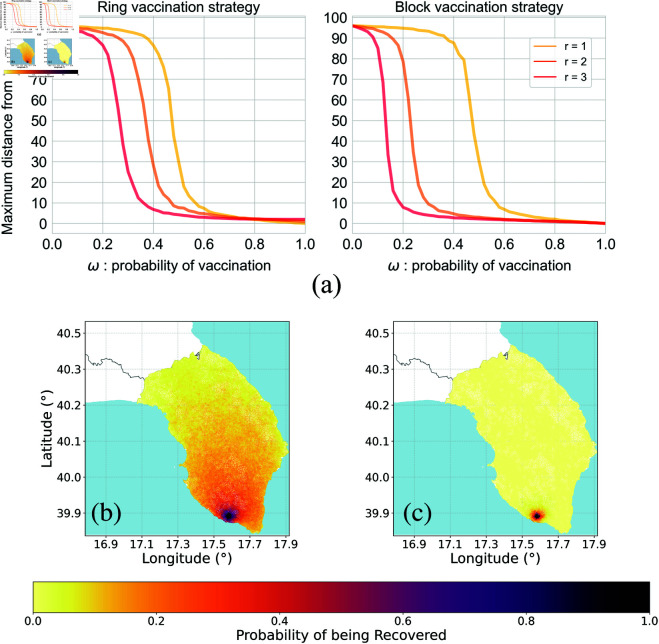
Impact of the vaccination strategy in the containment scenario applied to the empirical olive tree network. (A) displays the distance between the starting point of the epidemics and the farthest *R*-node, as a function of the probability of vaccination *ω*. Heatmaps (bottom) show the probability of each node to be in the recovered state at the end of the process, for, respectively, dynamic ring (B) and block (C) vaccination, for the case of ω=0.25. The epidemiological parameters are: probability of infection β=0.7, probability of recovery γ=1. Data for the creation of the maps taken from: https://www.istat.it/notizia/confini-delle-unita-amministrative-a-fini-statistici-al-1-gennaio-2018-2/.

To further characterize the spatial propagation of the disease, we address the question of the spatial reach of the epidemic. Assuming a fixed location as the starting point for the spread (known in this case, see the Method section for a detailed explanation), we investigate how the distance between the root node and the farthest *R*-node in the asymptotic state depends on various epidemiological parameters. For the containment vaccination scenario, we present in Fig 7 the evolution of such a distance as a function of the vaccination probability *ω* and for different radii *r*. For simplicity, we note that distance is not intended to have a geographical meaning; rather, we consider distance measured by the network’s shortest path.

Fig 7A shows that all curves exhibit similar behavior. When the vaccination probability is small, the disease can reach the entire system. However, as *ω* increases, the maximum distance from the root node sharply declines. Beyond this point, further increases in the vaccination probability lead to a gradual decline towards zero distance. Comparing the two containment vaccination strategies, it is immediately evident that block vaccination is significantly more effective at reducing the extent of the disease’s spatial propagation, especially for higher values of *r*. For *r* = 3, a vaccination probability of ω=0.2 results in a maximum distance of approximately 85 steps for the ring vaccination case, whereas the value is markedly lower in the block case, reaching about 10. This trend is further confirmed by examining the spatial risk maps in Fig 7B and 7C, where, for a fixed vaccination probability *ω*, we observe significantly lower probabilities of the disease spreading far from the initial root node in the block scenario.

## Discussion

Firstly, we focused on the preventive scenario, in which the vaccination is anticipated to prevent the epidemic from spreading that later unfolds on the network of non-vaccinated nodes. Hence, a crucial role is played by the heterogeneity of the contact patterns, both in block and ring vaccination. We verify this when comparing the vaccination strategies against the baseline of mass vaccination, see Fig 5. We observe increasing differences with respect to the baseline, in a way that the impact of the disease is decreased, as we go from more homogeneous to more heterogeneous topologies. In this preventive landscape, ring outperforms block vaccination in terms of dismantling the network in clusters that break the multiple potential chains of infection, thus reducing the outcome of the epidemic, *i.e.,* the asymptotic number of ever-infected individuals, the so-called recovered population. Furthermore, the vaccination radius *r* has little influence in the epidemic process: the values considered r∈[1,2,3] do not impact significantly either in the resulting network of non-vaccinated nodes (the percolation threshold $\phi_{c}$) nor in the asymptotic number of recovered individuals. The effects of *r* can be appreciated only for some cases in the ring Vaccination case, namely, for very high percentages of vaccinated nodes (ϕ≥0.5).

Secondly, we have focused on containment scenarios, in which the vaccination and the diffusion of the disease co-evolve. In this case, the nature of the problem is different, since infection and vaccination occur in similar time scales. Thanks to the proposed message-passing framework, we have access to the time evolution of the epidemic behavior. In this containment landscape, when we consider the same epidemic outcome, the general trend is that block outperforms ring vaccination in terms of the use of resources. In the case of the same asymptotic fraction of recovered individuals, fewer vaccines are needed in the case of block vaccination. Besides, here the radius of vaccination does play a remarkable role in reducing the impact of epidemic process. This is verified in Fig 5, where, for different values of *r*, the functional forms of the curves of the *SIR*–*V* model are significantly different. Consequently, the impact of the epidemic is strongly modulated by the radius chosen, always outperforming the baseline model in terms of the percentage of recovered nodes. Moreover, as we observed in Fig 5, *r* also plays an interesting role in characterizing the effectiveness of the process across the two strategies considered; particularly for *r* = 2 in which the block outperforms the ring strategy in terms of resources needed in order to have the same final outcome. Furthermore, in this dynamic vaccination case, we have introduced a parameter *ω* related to the vaccination probability. By focusing on the heatmaps presented in Fig 5, we learn that low values of the vaccination probability, such as ω=0.3, are enough to reduce the asymptotic population of recovered individuals, even for high values of the infectivity *β*. Moreover, for higher values of the vaccination parameter, almost no epidemics occur. Since *ω* can be interpreted as the successful identification of the contacts of infected individuals in a contact tracing procedure, this result is encouraging, because contact tracings with low coverage are still enough to prevent an epidemic, under the assumptions used here. Finally, we highlight that the vaccination technique chosen does not play a crucial role in this case: both block and ring do perform well Fig 5 in terms of reducing the advance of the diseases.

Given the complexity of the proposed model, a proper in-depth analysis of the role of the parameters is beyond the scope of the current work. Nevertheless, due to the realistic nature of these assumptions in the model, we dedicate a section in the Supporting Information to the case of: i) varying the recovery time of infected nodes, Fig F in S1 File; ii) varying the effectiveness of the vaccine Fig G in S1 File. A key finding is that lowering the recovery rate reduces the fraction of recovered individuals. This happens because infected nodes remain infectious longer, spreading the disease further. This is evident as the vaccinated curve for $\gamma =\frac{1}{3}$ is higher than for γ=1. Furthermore, Fig F in S1 File explores a scenario where vaccinated nodes can still be infected. When a node is vaccinated, it succeeds with probability λ=0.5; otherwise, it remains susceptible but is still counted as vaccinated. This distinction between “immune" and “non-immune" vaccinated individuals leads to a near disappearance of the percolation threshold, allowing epidemics even at low *β*. The epidemic impact is significantly higher, despite similar vaccination levels, as non-immune vaccinated individuals continue spreading the disease. Note how the proposed variation of the baseline models is just a preliminary and exploratory analysis of most real-life scenarios, for which an in-depth study is left to further work.

As a final point, we have applied the aforementioned vaccination models to an empirically motivated scenario by analyzing the diffusion of the epidemic and its containment in a spatial network. This has firstly allowed us to provide a visual representation of the evolving processes, focusing on how the spatial and topological correlation of such a network affects the outcome of theoretical models. We have then investigated spatial characteristics of the spreading, stressing how the reach of the epidemic is strongly influenced by the vaccination technique chosen (together with other parameters of the process), as exposed in Fig 7. Especially, for values of r≥1, block outperforms ring vaccination by strongly reducing the average distance of the farthest ever-infected node, also for small values of the vaccination probability *ω*. For instance, for *r* = 3, ω=0.2 is enough to prevent an outbreak in the block scenario. Finally, notice that, in order to deal with a network based on spatial properties, we focused on the Voronoi regions as the main theory to build our network. However, an interesting further analysis may come from exploring how the dynamic changes by considering the actual physical distance among trees: an alternative way of proceeding, indeed, is to consider the geographical distance between two trees as the weight of the corresponding edge, and retrieve the network by cutting all edges with a weight above a given threshold. This analysis is displayed in Fig E in S1 File, where we explore how different thresholds in the distance between trees lead to potentially different degree distributions. For the sake of simplicity, we did not explore these scenarios in the present work.

## Conclusion

When material and human resources are limited, efficient vaccination strategies are fundamental to curb transmission and reduce the impact of a disease outbreak. Here, we have focused on the mathematical and computational modeling of ring and block vaccination, a strategy that in the last decades has proven to be very useful to contain the spread of diseases, or even directly suppress them, such as in the case of smallpox [45]. Ring vaccination has been extensively adopted to contain the spread of the Ebola virus in the 2014-2016 West Africa outbreak [35,46], and later in the 2018 RDC outbreak [47]. More recently, ring vaccination has also been proposed as an effective strategy to contain the 2022-23 monkeypox virus (MPXV) outbreak [48,49]. The classic version of ring vaccination consists of immunizing the contacts of infected individuals to generate around them a herd immunity effect that breaks the chains of transmission. Hence, having reliable knowledge of the contact patterns of the population is central to the proper functioning of the strategy. However, due to a multitude of reasons, this knowledge may not always be completely accessible, putting at risk the very same principle that ring vaccination is based upon to perform efficiently. By recognizing that, when tracing contact patterns, some uncertainty may exist, and that immunizing contacts may not perfectly curb the spread, we need ways to tackle these elements when studying immunization strategies, in general, and ring vaccination, in particular.

We have addressed this problem by introducing a generalized family of ring vaccination strategies of arbitrary radius *r*. The case *r* = 1 corresponds to the classical case; while, for *r* > 1, two vaccination options can be implemented, resulting in very different management of available resources. On the one hand, within this generalized family of strategies, there is what we simply call *ring vaccination*, where vaccines are allocated on the corona of neighbors *r* hops away from an infected case. On the other hand, we have considered the so-called *block vaccination*, which immunizes all the neighbors up to radius *r*. These strategies have been studied in two further scenarios, corresponding to two limiting cases in the interplay between the time scales of vaccination and disease spreading. On one hand, the so-called static or preventive scenario is when all the vaccines are administrated before the disease reaches the population and propagates, as a preventive measure. Then, on top of the resulting network (with vaccinated and susceptible individuals), we simulate an epidemiological dynamics in order to test the effectiveness of the strategy. On the other hand, in the so-called dynamic or containment scenario, vaccination and disease spreading co-evolve in the same timescale, both phenomena are coupled.

Specific mathematical techniques have been tailored to study each vaccination strategy. For the preventive one, we have implemented an approach that combines teleporting self-avoiding random walks [50] and percolation theory, while for the containment one, we have employed a coupled dynamic message-passing approach. Our analytical approach has allowed us to obtain accurate estimates for the fraction of the population impacted by the disease. An efficient vaccination strategy minimizes the transmission chains, thus reducing the number of cases of infected individuals in the course of the outbreak. We have evaluated the effectiveness of each of the proposed strategies as a function of (i) the properties of the contact pattern network, (ii) the epidemiological parameters, and (iii) the vaccination radius *r*. Going beyond synthetic contact patterns, we have tested the effectiveness of the proposed vaccination strategies on an empirical network. Motivated by the recent outbreaks of the insect-borne pathogen *Xylella fastidiosa* in the Mediterranean basin, from the spatial distribution of olive trees in the Salento region (Italy), we have built a proximity-based tree network on top of which we have simulated a simplified epidemiological model. We have corroborated that the qualitative behavior found in synthetic topologies can be extended to empirical networks as well. Moreover, since in this case we work with a spatially embedded network, we have also characterized the interplay between the radial vaccination strategies and the spatial unfolding of the disease.

Our work offers insights into a family of vaccination strategies that can be useful when limited information and/or resources are present. It is based on the principle of creating immunization buffers to prevent global spreading. This is a first approach to modeling these scenarios and can be extended in multiple directions. An important aspect is to incorporate more realistic epidemiological models. This includes, for instance, models with more compartments such as those encompassing incubation periods, asymptomatic individuals, metapopulation, mobility, etc. In the case of the olive tree network, there exist epidemiological models [51,52] that take into account the vector-borne nature of the spreading of the *Xylella fastidiosa*, which certainly should be used if one aims to complement the qualitative understanding we offer here with quantitative predictions. Another dimension to explore, bridging the gap with more realistic modeling scenarios, is to consider the duration of the social interactions. This could be done by either considering temporal networks [53] as underlying structures or finding a way to continuously interpolate between our two extreme cases, the preventive (static) and containment (dynamic) vaccination scenarios. Similarly, one could explore the role played by multilayer [15] or higher-order [16] interactions. Moreover, these methods can also be applied in the context of online mis/disinformation spread, where vaccination can be seen as a way to inoculate individuals against false content. Having a clear understanding of all these aspects is necessary to reliably implement and test generalized ring vaccination strategies in empirical settings. We hope that our work here is a stepping stone toward that direction, and we advocate for steady progress in the mathematical modeling of epidemic spreading and vaccination scenarios to achieve this purpose.

## Methods

In this section, we provide the mathematical details to derive the equations displayed in the Results section. We first present the preventive scenario and later the containment one.

### Mathematical treatment of static vaccination

A vaccination event in the preventive scenario (static vaccination) is based on two steps. The first one is the selection and vaccination of a central node, while the second one is the vaccination of the neighborhood around that reference node, according to the block or ring strategy. The former is independent of the type of vaccination strategy, so we first discuss it, and later we particularize for each strategy.

As mentioned in the main text, we leverage percolation theory to address the static vaccination scenario, with vaccinated nodes in our epidemiological context playing the role of removed nodes in the percolation one [54]. Under this premise, we map the network structure in which the central node is selected (time *t*–1) and the one after that node is vaccinated/removed (time *t*). To do so, we adapt the results presented in [50], referring to this work for a precise derivation of the presented quantities. Focusing on the degree distribution, we obtain

$$p_{t}(k)=\frac{1}{N_{t}}\left[p_{t-1}(k)N_{t-1}-d_{t}(k)+\langle d\rangle_{t}\left[q_{t-1}(k)-q_{t-1}(k-1)\right]\right]$$ (14)

$$d_{t}(k)=p_{t-1}(k)$$ (15)

where *N*_*t*_ is the number of nodes in the network at time *t*, *q*_*t*_(*k*) is the time-dependent excess degree distribution, and *d*_*t*_ and $\langle d\rangle_{t}$ are, respectively, the time-dependent distribution of the degree of the central node vaccinated at time *t* and its expected value. The recursive Eqs (14) and (15) can easily be iterated numerically. The initial condition *N*_0_, *p*_0_ and *q*_0_ corresponds to the initial network size, the initial degree distribution and the initial excess degree distribution respectively.

Since the selection of the central node is made uniformly at random, we have $d_{t}(k)=p_{t-1}(k)$. Therefore, merging Eqs (14) and (15) yields Eq (1), presented in the main text. The reason to write separately Eqs (14) and (15) is to highlight that our analytical framework actually has plenty of freedom in choosing the central nodes for the block and ring vaccination. For instance, $d_{t}(k)=q_{t-1}(k)$ would select central nodes with a probability proportional to their degree, i.e., hubs would be chosen with much larger probabilities. Analysing the impact of the degree of the central node around which the block and rings unfold is certainly interesting, yet it falls outside the scope of this work, and we leave it as future research.

We move now to characterizing the network after the radial vaccination takes place. For the block vaccination case, we generalize the work of Shai and coauthors [41], where it is introduced a localized percolation strategy that removes (in our case, vaccinates) concentric neighborhoods of increasing distance around a root node. We need the expected number of *r*-distance neighbors of a node, namely the expected number of nodes at distance *r* from a target node. Let $\langle k\rangle_{t}$ and $\langle k^{2}\rangle_{t}$ be, respectively, the first and second moments of the probability distribution of the network at time *t*. Then, the average number of *r*-distance neighbors is [55]

$$c_{r,t}={\left(\frac{\langle k^{2}\rangle_{t}-\langle k\rangle_{t}}{\langle k\rangle_{t}}\right)}^{r-1}\cdot \langle k\rangle_{t}.$$ (16)

The percentage $\phi_{t}$ of nodes that will be vaccinated at time *t* is, then,

$$\phi_{t}=\frac{\Omega_{t}^{r}}{N_{t}},$$ (17)

where

$$\Omega_{t}^{r}=\sum_{i=1}^{r}c_{i,t}=\sum_{i=1}^{r}{\left(\frac{\langle k^{2}\rangle_{t}-\langle k\rangle_{t}}{\langle k\rangle_{t}}\right)}^{i-1}\cdot \langle k\rangle_{t}.$$ (18)

Referring to [41] for a detailed description of all the passages, the generating function of the degree distribution of the network after the block vaccination reads

$$G_{0}^{t+\Omega_{t}^{r}}(x)=\frac{1}{G_{0}^{t}(f)}G_{0}^{t}\left[f+\frac{\left(G_{0}^{t}{)}^{\prime }(f\right)}{\left(G_{0}^{t}{)}^{\prime }(1\right)}(x-1)\right],$$ (19)

where $f={\left(G^{t}\right)}_{0}^{-1}(\phi )$. Thus, one can easily find $p_{t+\Omega_{t}^{r}}(k)$ by inverting the relation $G_{0}^{t+\Omega_{t}^{r}}(z)=\sum_{k}p_{t+\Omega_{t}^{r}}(k)\cdot z^{k}$, which represents the input for Eqs (14) and (15).

In the ring vaccination scenario, we do not vaccinate all the neighbors up to a given radius but only those belonging to the specific shell *r*, together with the root node. In this way, all the other nodes belonging to the smaller shells are protected and not part of the giant component anymore, nonetheless, they remain active and in the network.

To model mathematically this scenario, we consider two distinct phases. In the first phase, all nodes up to the radius *r* are virtually vaccinated similarly to the block case, and the degree probability distribution is updated through its generating function (19). We have now two probability distributions, *p*_*t*_(*k*) and $p_{t^{\prime }}(k)$, where $t^{\prime }=t\;+\;\Omega_{t}^{r}$ is the time we would move to by applying the block rules. What we need to do now is to “reintroduce” in the network the nodes belonging to the inner shells. At practical effects, this is like unvacinnate the nodes that do not belong to the corona. This number of nodes amounts to

$$\Omega_{t}^{r-1}=\sum_{i=1}^{r-1}c_{i,t}=\sum_{i=1}^{r-1}{\left(\frac{\langle k^{2}\rangle_{t}-\langle k\rangle_{t}}{\langle k\rangle_{t}}\right)}^{i-1}\cdot \langle k\rangle_{t}.$$ (20)

Hence, the effective elapsed time, i.e., the actual number of vaccinated nodes in the ring case is given by $\Delta \Omega_{t}\equiv \Omega_{t}^{r}-\sum_{i=1}^{r-1}\Omega_{t}^{i}$.

We are free to choose how the nodes of the internal shells are reinserted. Ideally, they should keep their actual topology without the links that point towards the central node and its *r*-neighbors, yet this complicates the mathematical treatment. Here we opt for a simpler approach which, nevertheless, turns out to approximate well the metrics we are interested in. Our choice is to make the strong assumption that the reinserted nodes do so with degree 0, i.e., they are disconnected. If we denote by $n_{k}^{t}$ the number of nodes with degree *k* at time *t*, then

$$n_{k}^{t+\Delta \Omega_{t}}=\left\{ \begin{array}{cc} p^{t^{\prime }}(k)\cdot N_{t^{\prime }}, & k>0\\ p^{t^{\prime }}(k)\cdot N_{t^{\prime }}+\Omega_{t}^{r-1}, & k=0, \end{array}\right.$$ (21)

and the new degree distribution after the ring vaccination event is, simply,

$$p^{t+\Delta \Omega_{t}}(k)=\frac{n_{k}^{t+\Delta \Omega_{t}}}{\sum_{i=1}^{k_{max}}n_{i}^{t+\Delta \Omega_{t}}}.$$ (22)

This approximation will fail if the vaccination radius *r* becomes very large. We have checked that the agreement between theory and simulations is still good up to *r* = 4. However, since the average path length of uncorrelated networks goes as $\frac{\ln(\frac{N}{\langle k\rangle })}{\ln(\frac{\langle k^{2}\rangle }{\langle k\rangle })}$, the cases of large *r* would correspond to huge networks for which other problem would anyway arise, such as the possibility of having a reliable contract tracing.

As already stated in the Results section, a key quantity we focus on to quantify the effectiveness of the proposed strategy is the size of the time-dependent giant component *s*_*t*_. Since the giant component of a network can be defined as a connected component which contains a relevant proportion of the nodes in a network, being able to dismantle such component is fundamental in order to avoid an epidemic to emerge. Once the degree distribution or the generating function are available, obtaining the time-dependent normalized size of the giant component *s*_*t*_ is trivial. We need to solve the system of equations [50,55]

$$\left\{ \begin{array}{c} u_{t}=G_{1}^{t}(u_{t})\\ s_{t}=1-G_{0}^{t}(u_{t}) \end{array}\right.$$ (23)

where $G_{1}^{t}(u_{t})$ is the time-dependent excess degree generating functions of the unvaccinated nodes. *s*_*t*_ represents an upper bound of the outbreak size for a disease with very large infectivity, defined as the fraction of recovered nodes *R* in the main text. In general, though, $R<\max_{t}(s_{t})$. To find the actual value *R*, which is used to quantify the effectiveness of the vaccination strategy, we exploit the mapping between bond percolation and epidemics, which make use of the degree distributions and generating functions that we have already obtained [19].

### Mathematical treatment of dynamic vaccination

In the containment scenario, vaccination takes place simultaneous to the spreading of the epidemic. Nodes can be in any of 4 states, namely, susceptible (*S*), infected (*I*), recovered (*R*) and vaccinated (*V*). We take a node-centered approach, and for each node *i* we monitor the probabilities that, at time *t*, node *i* is in state $q_{i}^{X}(t)$, with X∈{S,I,R,V}. Time *t* is discrete and at each step *t* nodes try to update their state or the one of the neighboring nodes according to the current selection of vaccination models and parameters. As initial condition, we take a given low percentage of nodes as infected, while all the rest of the elements are susceptible. To obtain the equations for $q_{i}^{X}(t)$, message passing techniques [56] are exploited and adapted.

The model transitions are S→I, i.e., a susceptible node becomes infected by an infected 1-neighbor with probability *β*, I→R, i.e., infected nodes spontaneously recover with probability *γ*. Finally, we have S→V, i.e., a susceptible node is vaccinated with probability *ω* due to the influence of an infected *r*-neighbor in the ring case or of an infected $r^{\prime }$-neighbor, with $r^{\prime }=1,\ldots ,r$, in the block case.

We define two fundamental quantities for the message passing treatment: $\theta_{k\to i}(t)$ and $\delta_{k\to i}(t)$, respectively, referring to the probability that the infection and the vaccination messages have not been passed from node *k* to node *i* up to time *t*. Recursively, these two quantities can be written as

$$\theta_{k\to i}(t+1)=\theta_{k\to i}(t)-\beta \cdot \phi_{k\to i}(t)\;\delta_{k\to i}(t+1)=\delta_{k\to i}(t)-\omega \cdot \phi_{k\to i}(t),$$ (24)

where $\phi_{k\to i}(t)$ refers to the probability that no message passed from node *k* to node *i* up to time *t* and node *k* is infected, so it can pass the message to node *i*. Eqs (24) are to be interpreted as the probabilities that a given message has not been passed between two nodes up to time t+1 equal the probability that the message has not been passed up to time *t*, minus the probability that the two nodes, at time *t*, are in the condition to make possible the message passing ($\phi_{k\to i}(t)$) times the probability that the message passes - respectively *β* and *ω*.

For the ring vaccination case, we compute $\phi_{k\to i}(t)$ recursively such that

$$\phi_{k\to i}(t+1)=\phi_{k\to i}(t)\cdot (1-\beta )\cdot (1-\gamma )\cdot (1-\omega )\;+q_{k\to i}^{S}(0)\cdot \Delta \theta_{j\to k}(t+1)\cdot \prod_{j\in \partial^{r}k⧵i}\delta_{j\to k}(t+1),$$ (25)

with $\Delta \theta_{j\to k}(t\;+\;1)\equiv \prod_{j\in \partial k⧵i}\theta_{j\to k}(t)\;-\;\prod_{j\in \partial k⧵i}\theta_{j\to k}(t\;+\;1)$. The right-hand side of Eq (25) is composed of two different terms. The first term represents the probability that nodes *k* and *i* are capable of passing the message up to time *t* and no change occurred between *t* and t+1. The second one refers to the probability that node *k* was susceptible up to time *t* and becomes infected at time t+1. The latter is obtained as the product of three different factors: (i) $q_{k}^{S}(0)$, the probability that node *k* was susceptible at time *t* = 0, (ii) $\Delta \theta_{j\to k}(t\;+\;1)$, the probability that no infection message passed between each neighbor of *k* and *k* up to time *t*, and hence the difference refers to the probability that the infection message passes between *t* and t+1, and, (iii) the probability that no vaccination occurs between any of the *r*-neighbors of *k* and *k* up to time t+1, corresponding to the products of $\theta_{j\to k}$. Note that this last point is actually the one encoding the ring condition of the vaccination, and it will be modified when assessing the block case.

With Eqs (24) and (25) at hand, we are in the position of obtaining the temporal evolution of the probabilities of a node belonging in each of the possible states for the ring vaccination case. They are given by

$$q_{i}^{S}(t)=q_{i}^{S}(0)\cdot \prod_{k\in \partial i}\theta_{k\to i}(t)\cdot \prod_{k\in \partial^{r}i}\delta_{k\to i}(t)$$ (26)

$$q_{i}^{I}(t)=q_{i}^{I}(t-1)\cdot (1-\gamma )+q_{i}^{S}(0)\cdot \Delta \theta_{k\to i}(t)\cdot \prod_{k\in \partial^{r}i}\delta_{k\to i}(t)$$ (27)

$$q_{i}^{R}(t)=q_{i}^{R}(t-1)+q_{i}^{I}(t-1)\cdot \gamma$$ (28)

$$q_{i}^{V}(t)=1-q_{i}^{S}(t)-q_{i}^{I}(t)-q_{i}^{R}(t).$$ (29)

Notice that the term $\prod_{j\in \partial^{r}k⧵i}\delta_{j\to k}(t\;+\;1)$ refers to the probability that no vaccination occurs between any of the *r*-neighbors of *k* and *k* itself up to time t+1. To adapt to the block vaccination strategy, we seek for the probability that no vaccination occurs between any of the *i*-neighbors of *k* and *k*, for i=1,…,r. Similarly, the last term in Eqs (26) and (27) need to be completed with all the other multiplicative terms referring to the other shells that now do contribute to the potential vaccination. We can hence rewrite Eq (25) as


$$\phi_{k\to i}(t+1)=\phi_{k\to i}(t)\cdot (1-\beta )\cdot (1-\gamma )\cdot (1-\omega )\;+q_{k\to i}^{S}(0)\cdot \Delta \theta_{j\to k}(t+1)\cdot \prod_{\rho =1}^{r}\left(\prod_{j\in \partial^{\rho }k⧵i}\delta_{j\to k}(t+1)\right),$$

and, similarly, Eqs (26) and (27) as

$$q_{i}^{S}(t)=q_{i}^{S}(0)\cdot \prod_{k\in \partial i}\theta_{k\to i}(t)\cdot \prod_{\rho =1}^{r}\left(\prod_{k\in \partial^{\rho }i}\delta_{k\to i}(t)\right)$$ (30)

$$q_{i}^{I}(t)=q_{i}^{I}(t-1)\cdot (1-\gamma )+q_{i}^{S}(0)\cdot \Delta \theta_{k\to i}(t)\cdot \prod_{\rho =1}^{r}\left(\prod_{k\in \partial^{\rho }i}\delta_{k\to i}(t)\right)$$ (31)

The temporal evolution of the probability of being in the recovered and the vaccinated state remain the same as in the ring vaccination case.

### Synthetic networks

Three different network families are considered throughout the work. They are, from more homogeneous to more heterogeneous, the random regular graphs (RR), Erdős–Rényi (ER) and Scale-Free (SF) networks. Their degree distributions are $p^{\left(RR\right)}(k)=\delta_{k,\hat{k}}$, *i.e.*, all nodes have the same degree $\hat{k}$, $p^{\left(ER\right)}(k)\approx e^{-\langle k\rangle }\frac{{\langle k\rangle }^{k}}{k!}$, and $p^{\left(SF\right)}(k)\propto k^{-\gamma }$ with $k\in \left[k_{\text{min}},\ldots ,k_{\text{max}}\right]$.

All networks are obtained through the Configuration Model, a method that allows one to generate random graphs starting from a given degree distribution. Note that the topological parameters exposed above play a crucial role in the evolution of epidemic scenarios on networks. Different values of ⟨k⟩ and *γ* produce variations in the threshold value for the rise of the epidemic and, more generally, modify the inner structure of the network itself.

Finally, stochastic simulations of the defined techniques on the proposed synthetic networks are performed. We average over 10^3^ simulations for each vaccination/diffusion process, and compute the quantities that characterize each outcome (final size of the epidemic in the preventive scenario, percentages of individuals belonging to each state in the containment one). In all the cases presented, the size of the points is larger than the corresponding error bars, and, for this reason, they are not displayed.

### Empirical network

We analyze a network obtained from a geotagged dataset containing the exact position of 61 036 olive groves in the Apulia region, in Italy. The original shapefile was obtained directly from G. Strona, one of the authors of Ref [42]. From this, we filtered the dataset in order to keep only those groves belonging to Lecce province. Then, the correspondent network was built by calculating the Voronoi diagram of the spatial data: each grove is linked to a correspondent unique Voronoi region, and a link between two groves exists if the correspondent Voronoi regions share a common border. The result is a network formed by 13 306 nodes and 39 553 edges, with ⟨k⟩=5.95. There is a historic reason behind the choice of such an empirical network: during the last ten years, olive trees in the southern part of the Apulia region suffered from a strong epidemic of *Xylella Fastidiosa*, a bacterium that spread through the majority of trees in the subregion, moving to the north and threatening the olive production through Italy [51]. Since the initial position of the first outbreak has been identified [57] to be somewhere close to the town of Gallipoli, we have decided to set a random olive grove in that municipality as the starting node for the evolution of the model. Hence, differently than what happens for synthetic networks, here the diffusion of the disease will always start from the same area.

## Supporting information

S1 FileAppendix.(PDF)**Fig A. Evolution of the time-dependent degree distribution *p*_*t*_(*k*) for an ER network under block (top) and ring (bottom) vaccination in preventive scenario.** Each line represents a different percentage of vaccinated individuals at the end of the process. Different figures refer to different values of *r*, from 1 to 3 from left to right.(PDF)**Fig B. Impact of Block vaccination strategy for a preventive scenario on the olive tree empirical network: evolution of the diseases.** Screenshots of the advance of the diseases at different times, spanning from the early stages of the propagation (a) to the asymptotic state when there is no more presence of infected nodes (c). Single nodes are colored according to their state during the process: Susceptible (green), Infected (red), Recovered (gray), and Vaccinated (blue). The epidemiological parameters values are the probability of infection β=1, the probability of recovery γ=1, and the probability of vaccination ω=0.2. Data for the creation of the maps taken from: https://www.istat.it/notizia/confini-delle-unita-amministrative-a-fini-statistici-al-1-gennaio-2018-2/.(PDF)**Fig C. Impact of Ring vaccination strategy for a preventive scenario on the olive tree empirical network: evolution of the diseases.** Screenshots of the advance of the diseases at different times, spanning from the early stages of the propagation (a) to the asymptotic state when there is no more presence of infected nodes (c). Single nodes are colored according to their state during the process: Susceptible (green), Infected (red), Recovered (gray), and Vaccinated (blue). The epidemiological parameters values are the probability of infection β=1, the probability of recovery γ=1, and the probability of vaccination ω=0.2. Data for the creation of the maps taken from: https://www.istat.it/notizia/confini-delle-unita-amministrative-a-fini-statistici-al-1-gennaio-2018-2/.(PDF)**Fig D. Impact of Ring Vaccination strategy for a containment scenario on the olive tree empirical network: evolution of the diseases.** Screenshots of the advance of the diseases at different times, spanning from the early stages of the propagation (a) to the asymptotic state when there is no more presence of infected nodes (c). Single nodes are colored according to their state during the process: Susceptible (green), Infected (red), Recovered (gray), and Vaccinated (blue). The epidemiological parameters values are the probability of infection β=1, the probability of recovery γ=1, and the probability of vaccination ω=0.2. Data for the creation of the maps taken from: https://www.istat.it/notizia/confini-delle-unita-amministrative-a-fini-statistici-al-1-gennaio-2018-2/.(PDF)**Fig E. Analysis of key quantities of the geographical tree networks. (a) Degree distribution of the empirical networks.** Dotted green line represents the degree distribution of the original empirical network, obtained by mapping each tree to the correspondent Voronoi region and creating edges between nodes if the two correspondent Voronoi regions share a common border. Straight lines represent the degree distribution of the tree network with edges depending on the proper geographical distance between trees. Each value stands for a different threshold in meters. (b) Evolution of the size of the giant component. For each threshold, the size of the giant component of the resultant network is computed. (c) Evolution of the epidemic for the geographical tree network with threshold = 1000m under ring vaccination in the containment scenario. Two different values of the vaccination probability *ω* are considered. Other parameters of the process are γ=1, *r* = 2.(PDF)**Fig F. Epidemic impact in the containment scenario.** We consider scale-free networks with an exponent γ=2.5, $k_{\text{min}}=3$ and $k_{\text{max}}=20$, and size *N* = 10^4^. The recovery probability is set in $\gamma =\frac{1}{3}$, vaccination radius *r* = 2. Each line corresponds to different values of vaccination probability ω=0.15,0.25,0.35.. Top: Evolution of the fraction of recovered individuals. Bottom: Evolution of the fraction of vaccinated individuals. Figures on the left refer to block vaccination, figures on the right refer to ring vaccination.(PDF)**S1 Fig G. Epidemic impact in the containment scenario.** We consider scale-free networks with an exponent γ=2.5, $k_{\text{min}}=3$ and $k_{\text{max}}=20$, and size *N* = 10^4^. The recovery probability is set in γ=1, the vaccination efficacy probability λ=0.5, while vaccination radius *r* is set equal to 2. Each line corresponds to different values of vaccination probability ω=0.15,0.25,0.35. Top: Evolution of the fraction of recovered individuals. Bottom: Evolution of the fraction of vaccinated individuals. Figures on the left refer to block vaccination, figures on the right refer to ring vaccination.(PDF)
