# Supplementary material for: Comparing the effectiveness of ring and block-vaccination strategies on networks
Source: PLoS Comput Biol. 2025 Aug 11;21(8):e1013274. doi: 10.1371/journal.pcbi.1013274 (PMC12338826; doi:10.1371/journal.pcbi.1013274)
Supplement: S1 File — (PDF) Fig A. Evolution of the time-dependent degree distribution pt(k) for an ER network under block (top) and ring (bottom) vaccination in preventive scenario. Each line represents a different percentage of vaccinated individuals at the end of the process. Different figures refer to different values of r, from 1 to 3 from left to right. (PDF) Fig B. Impact of Block vaccination strategy for a preventive scenario on the olive tree empirical network: evolution of the diseases. Screenshots of the advance of the diseases at different times, spanning from the early stages of the propagation (a) to the asymptotic state when there is no more presence of infected nodes (c). Single nodes are colored according to their state during the process: Susceptible (green), Infected (red), Recovered (gray), and Vaccinated (blue). The epidemiological parameters values are the probability of infection β=1, the probability of recovery γ=1, and the probability of vaccination ω=0.2. Data for the creation of the maps taken from: https://www.istat.it/notizia/confini-delle-unita-amministrative-a-fini-statistici-al-1-gennaio-2018-2/. (PDF) Fig C. Impact of Ring vaccination strategy for a preventive scenario on the olive tree empirical network: evolution of the diseases. Screenshots of the advance of the diseases at different times, spanning from the early stages of the propagation (a) to the asymptotic state when there is no more presence of infected nodes (c). Single nodes are colored according to their state during the process: Susceptible (green), Infected (red), Recovered (gray), and Vaccinated (blue). The epidemiological parameters values are the probability of infection β=1, the probability of recovery γ=1, and the probability of vaccination ω=0.2. Data for the creation of the maps taken from: https://www.istat.it/notizia/confini-delle-unita-amministrative-a-fini-statistici-al-1-gennaio-2018-2/. (PDF) Fig D. Impact of Ring Vaccination strategy for a containment scenario on the olive tree [file pcbi.1013274.s001.pdf]

Supporting Information

In this section, we provide some supporting figures for the results discussed in the main paper.

Evolution of the time-dependent degree distribution

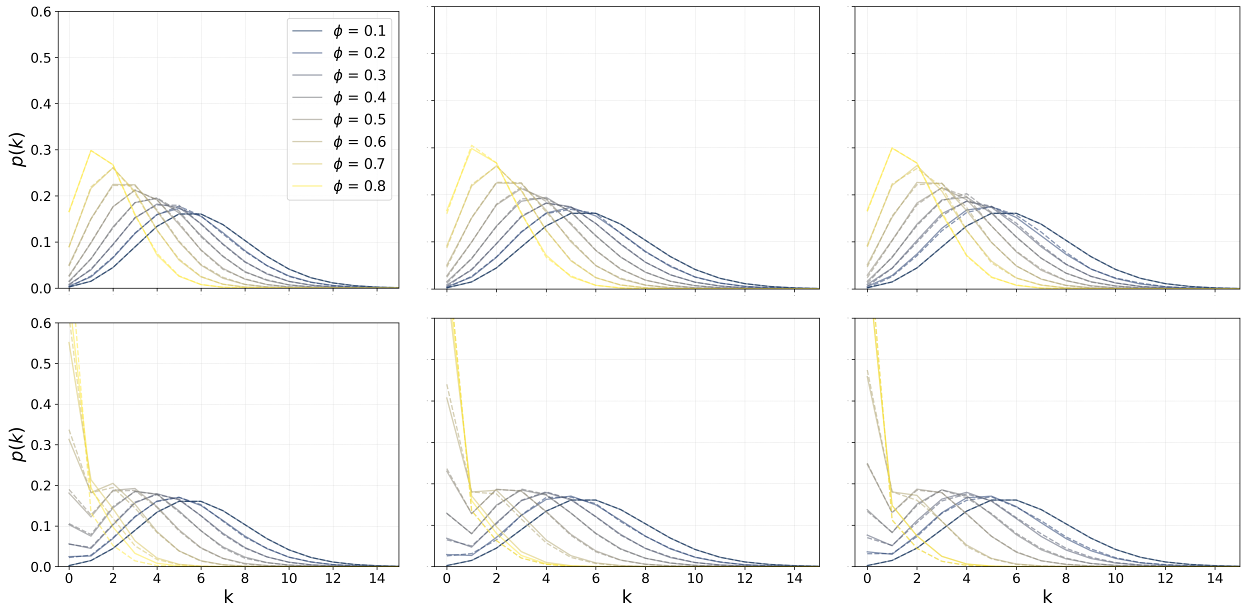

Fig A. **Evolution of the time-dependent degree distribution $p_t(k)$ for an ER network under block (top) and ring (bottom) vaccination in preventive scenario**. Each line represents a different percentage of vaccinated individuals at the end of the process. Different figures refer to different values of r, from 1 to 3 from left to right.

Empirical case of study: vaccination strategies apply to the olive tree network

Preventive scenario

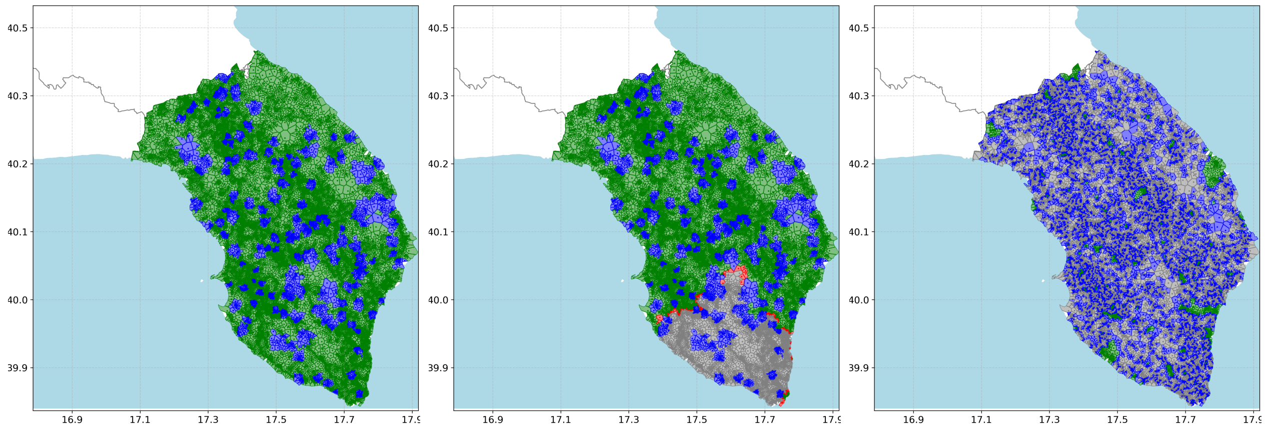

Fig B. **Impact of Block vaccination strategy for a preventive scenario on the olive tree empirical network: evolution of the diseases**. Screenshots of the advance of the diseases at different times, spanning from the early stages of the propagation (a) to the asymptotic state when there is no more presence of infected nodes (c). Single nodes are colored according to their state during the process: Susceptible (green), Infected (red), Recovered (gray), and Vaccinated (blue). The epidemiological parameters values are the probability of infection $\beta = 1$, the probability of recovery $\gamma = 1$, and the probability of vaccination $\omega = 0.2$. Data for the creation of the maps taken from: https://www.istat.it/notizia/confini-delle-unita-amministrative-a-fini-statistici-al-1-gennaio-2018-2/

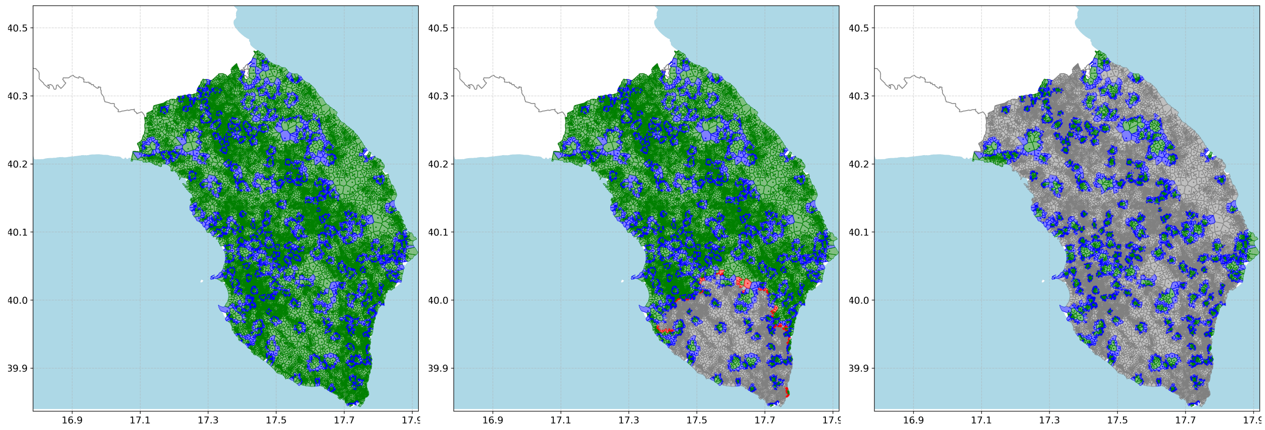

Fig C. **Impact of Ring vaccination strategy for a preventive scenario on the olive tree empirical network: evolution of the diseases**. Screenshots of the advance of the diseases at different times, spanning from the early stages of the propagation (a) to the asymptotic state when there is no more presence of infected nodes (c). Single nodes are colored according to their state during the process: Susceptible (green), Infected (red), Recovered (gray), and Vaccinated (blue). The epidemiological parameters values are the probability of infection $\beta = 1$, the probability of recovery $\gamma = 1$, and the probability of vaccination $\omega = 0.2$. Data for the creation of the maps taken from: https://www.istat.it/notizia/confini-delle-unita-amministrative-a-fini-statistici-al-1-gennaio-2018-2/

Containment scenario

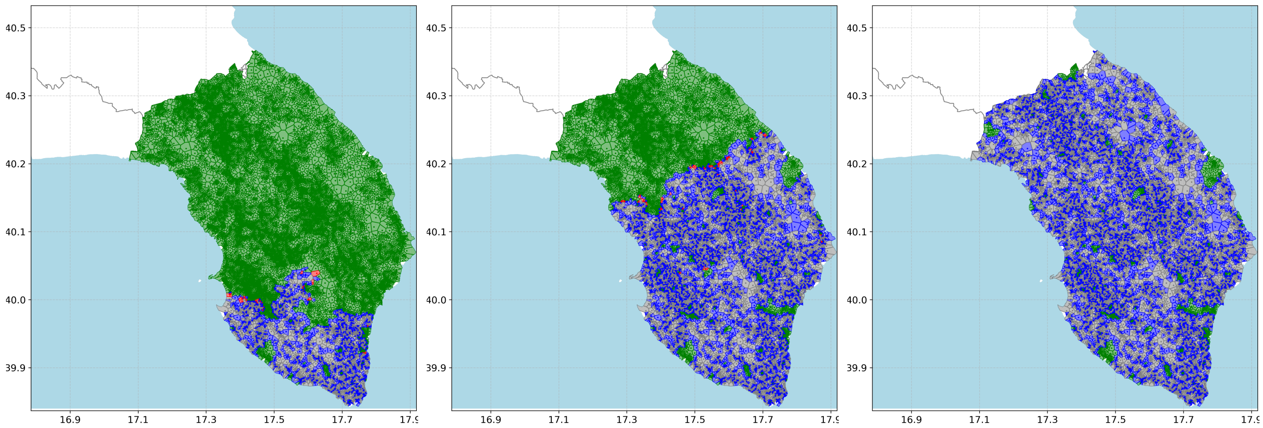

Fig D. **Impact of Ring Vaccination strategy for a containment scenario on the olive tree empirical network: evolution of the diseases**. Screenshots of the advance of the diseases at different times, spanning from the early stages of the propagation (a) to the asymptotic state when there is no more presence of infected nodes (c). Single nodes are colored according to their state during the process: Susceptible (green), Infected (red), Recovered (gray), and Vaccinated (blue). The epidemiological parameters values are the probability of infection $\beta = 1$, the probability of recovery $\gamma = 1$, and the probability of vaccination $\omega = 0.2$. Data for the creation of the maps taken from: https://www.istat.it/notizia/confini-delle-unita-amministrative-a-fini-statistici-al-1-gennaio-2018-2/

Exploration of empirical network structure

We focus here on some variation of the definition of the empirical olive trees network. Specifically, we leverage on the actual geographical distance between trees in order to define the existence of edges among different nodes. Relevant results are exposed in Fig A-B-C in SE. As we can see, both the probability degree distribution p_k and the size of the giant component strongly depend on the choice of the threshold for the distance between two neighbors.

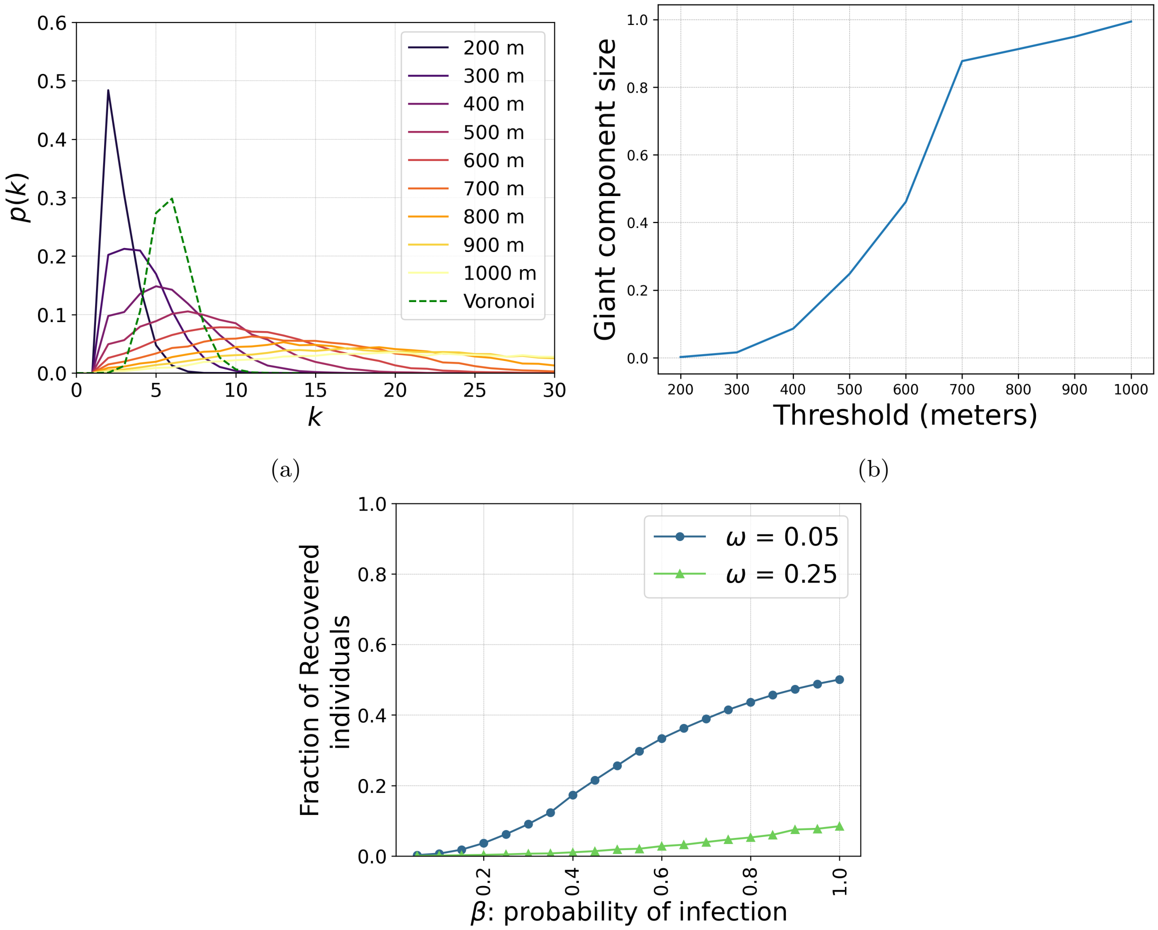

(a) (b)

(c)

Fig E. **Analysis of key quantities of the geographical tree networks**. (a) Degree distribution of the empirical networks. Dotted green line represents the degree distribution of the original empirical network, obtained by mapping each tree to the correspondent Voronoi region and creating edges between nodes if the two correspondent Voronoi regions share a common border. Straight lines represent the degree distribution of the tree network with edges depending on the proper geographical distance between trees. Each value stands for a different threshold in meters. (b) Evolution of the size of the giant component. For each threshold, the size of the giant component of the resultant network is computed. (c) Evolution of the epidemic for the geographical tree network with threshold = 1000m under ring vaccination in the containment scenario. Two different values of the vaccination probability ω are considered. Other parameters of the process are $\gamma = 1$, $r = 2$.

Further analysis on the parameters setting

We focus now on some variations of the proposed models, aiming to a more realistic representation of the considered scenarios. Note how these variations of the baseline models are just a preliminary and exploratory analysis of most real-life scenarios, for which an in depth study is left to further works.

Recovery rate

Differently than before, where the recovery rate γ was always set equal to 1, leading to an immediate recover at time $t + 1$ for all the nodes infected at time t, we now set $\gamma = \frac{1}{3}$. The first remarkable result from Fig SF is that reducing the recovery rate actually reduces the fraction of recovered individuals too. This can be derived from the fact that the infected nodes can in fact continue to spread the vaccination to more distant nodes than

those that can contract the disease from the same infected individual. This fact can also be corroborated by highlighting how the curve of vaccinated individuals, in the case of $\gamma = \frac{1}{3}$, is indeed higher than the correspondent curve at baseline $\gamma = 1$.

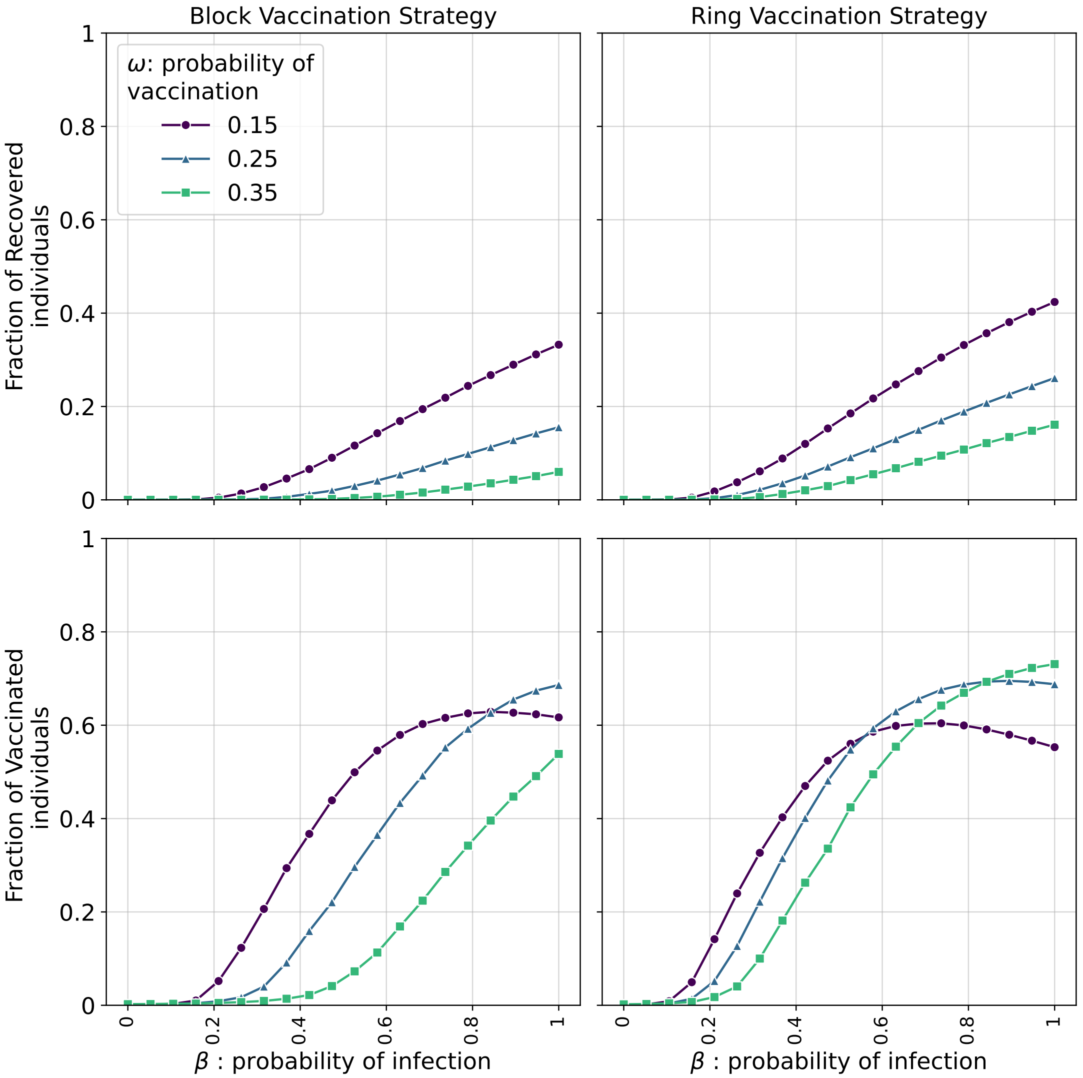

Fig F. **Epidemic impact in the containment scenario**. We consider scale-free networks with an exponent $\gamma = 2.5$, $k_{\min} = 3$ and $k_{\max} = 20$, and size $N = 10^4$. The recovery probability is set in $\gamma = \frac{1}{3}$, vaccination radius $r = 2$. Each line corresponds to different values of vaccination probability $\omega = 0.15, 0.25, 0.35$.. Top: Evolution of the fraction of recovered individuals. Bottom: Evolution of the fraction of vaccinated individuals. Figures on the left refer to block vaccination, figures on the right refer to ring vaccination.

Vaccination efficacy

We focus now on a news setting, as exposed in Fig SG, where vaccinated nodes can still get the disease by infected neighbors. Specifically, when a node is attempted to be vaccinated, it succeeds with probability λ (here set equal to 0.5). Otherwise, the individual is still seen as vaccinated from the system, but it actually can still get the infection and pass it to its susceptible neighbors. This allows us to distinguish, inside the Vaccinated status, between "immune vaccinated" individuals (succesfully vaccinated) and "not-immune vaccinated" ones (which can still spread the disease). We can see how, in this case, the percolation threshold almost disappear and an epidemic occurs for even very low values of β. Furthermore, the impact of the epidemic is way higher

in this case, with almost the same percentages of vaccinated individuals. This is coherent with the theory proposed, since even if both immune and non-immune vaccinated individuals are counted as vaccinated, the latter can though still contribute in the disease spreading leading to higher final impact of the epidemic.

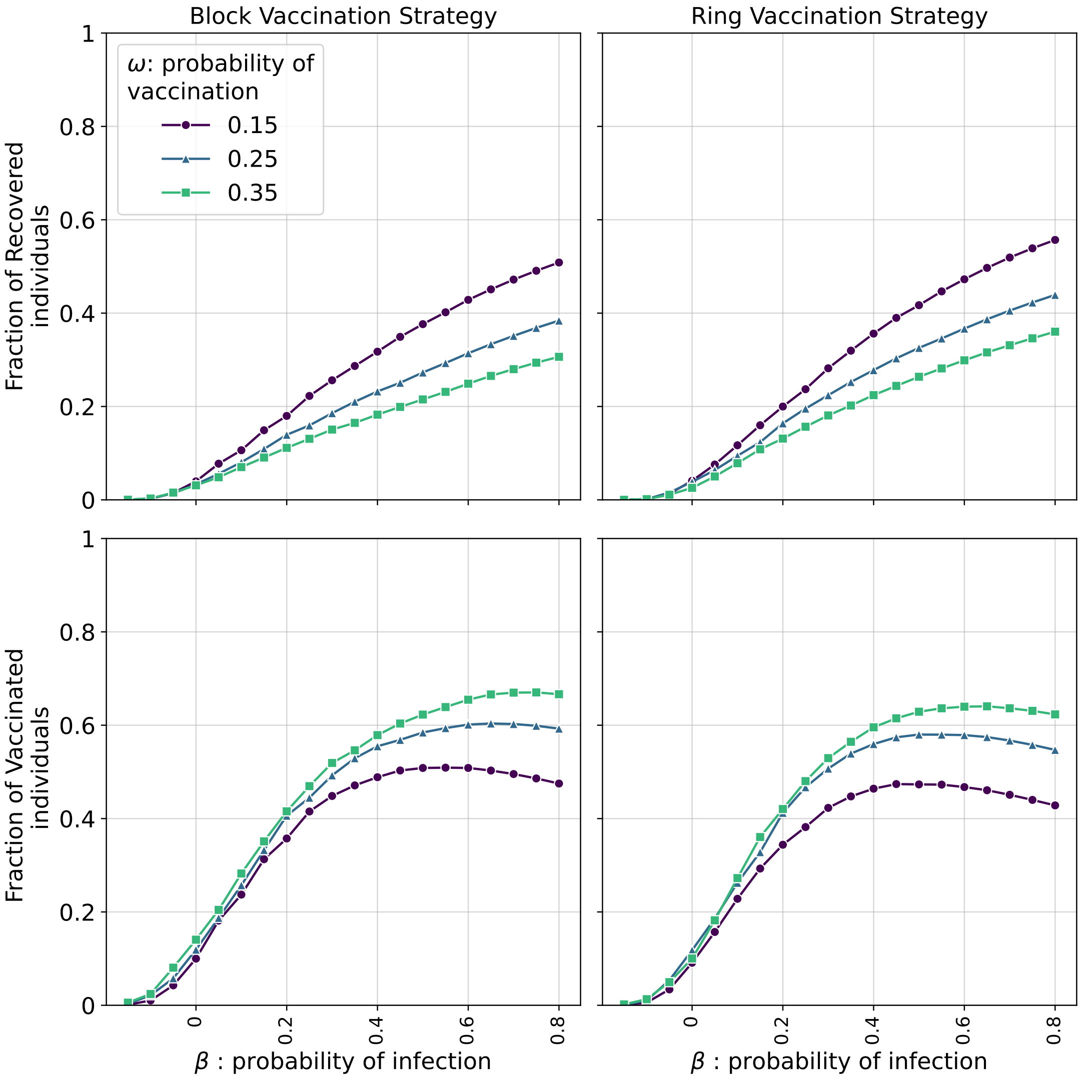

Fig G. **Epidemic impact in the containment scenario**. We consider scale-free networks with an exponent $\gamma = 2.5$, $k_{\min} = 3$ and $k_{\max} = 20$, and size $N = 10^4$. The recovery probability is set in $\gamma = 1$, the vaccination efficacy probability $\lambda = 0.5$, while vaccination radius r is set equal to 2. Each line corresponds to different values of vaccination probability $\omega = 0.15, 0.25, 0.35$. Top: Evolution of the fraction of recovered individuals. Bottom: Evolution of the fraction of vaccinated individuals. Figures on the left refer to block vaccination, figures on the right refer to ring vaccination.
